# Supplementary material for: Remote Sensing Provides a Rapid Epidemiological Context for the Control of African Swine Fever in Germany
Source: Sensors (Basel). 2023 Sep 30;23(19):8202. doi: 10.3390/s23198202 (PMC10575123; doi:10.3390/s23198202)
Supplement: Supplementary file 1 [file sensors-23-08202-s001.zip › Supplementary File S1.pdf]

Supplementary Information – Questionnaire

Bergmann et al. - “Remote sensing provides a rapid epidemiological context for the control of African swine fever in Germany”

GERMAN (original language, as administered):

In welchem Bereich sind Sie bei der ASP Bekämpfung in Deutschland involviert?

- auf Landesebene
- auf Kreisebene

Fernerkundungsdaten für die Bekämpfung der Afrikanischen Schweinepest: Inwiefern haben Sie bei der Maßnahmenplanung die Fernerkundungsdaten zu den Maisfeldern, dem Erntestatus der Maisfelder sowie der Eichen- und Buchen-Anteile an Waldflächen verwendet? Bitte wählen Sie aus.

(Radiobutton: Antwortmöglichkeiten für die folgenden Anwendungen der Fernerkundungsdaten)

- Nicht verwendet
- verwendet, aber keine Beeinflussung
- verwendet, mit geringfügiger Beeinflussung
- verwendet mit maßgeblicher Beeinflussung

Planung der Suche nach Wildschweinkadavern (Festlegung des abzusuchenden Areals)

Planung der Suche nach Wildschweinkadavern (Festlegung der Suchmethode, z.B. Drohne, Menschen usw.)

Planung nachfolgender Suchen nach Wildschweinkadavern (2. Suche usw.)

Planung von ASP-Kontrollzäunen (Festlegung des Zaunverlaufs)

Überprüfung der Einhaltung des Ernteverbots für Mais

ENGLISH (translation of the original questionnaire, as administered):

At what jurisdictional level are you involved with ASF control in Germany?

- at state level
- at district level

Remote sensing data for African swine fever control: To what extent did you use remote sensing data on maize fields, maize field harvest status, as well as oak and beech forest distributions in your planning of control measures? Please select.

(Radio button: Response options for the following remote sensing data applications.)

- not used
- used, but no influence
- used, with minor influence
- used with major influence

Selection of area for wild boar carcass searches (Determination of the area to be searched)

Selection of method for wild boar carcass searches (Determining the search method, e.g. drone, people, etc.)

Selection of area for follow up wild boar carcass searches (2. search etc.)

Positioning of wild boar fencing (Determination of the course of the fence line)

Compliance audit of maize harvesting ban
